# Asiaticoside Suppresses Gastric Cancer Progression and Induces Endoplasmic Reticulum Stress through the miR-635/HMGA1 Axis

**DOI:** 10.1155/2022/1917585

**Published:** 2022-06-02

**Authors:** Chao Zhang, Xiaolin Ji, Zhenguang Chen, Zhichao Yao

**Affiliations:** ^1^Department of General Surgery, The Second Affiliated Hospital of Wenzhou Medical University, Wenzhou, 325000 Zhejiang, China; ^2^Wenzhou Medical University, Wenzhou, 325035 Zhejiang, China; ^3^Department of Gastrointestinal Surgery, Shulan (Hangzhou) Hospital, Hangzhou, 310004 Zhejiang, China

## Abstract

**Objective:**

Gastric cancer is a prevalent malignant tumor with high morbidity and poor prognosis. Asiaticoside (AC) has antitumor effects, while its role in gastric cancer is elusive. Thus, this study investigated the effect of AC on gastric cancer progression.

**Methods:**

Cell viability and migration were determined using the CCK-8 and Transwell migration assay. Endoplasmic reticulum stress was detected through measuring the expressions of GRP78, Chop, and hnRNPA1 by Western blot. The luciferase assay confirmed the relationship between miR-635 and High Mobility Group AT-Hook 1 (HMGA1). The effect of AC on tumor growth was evaluated by establishing a xenograft tumor. The survival rate of mice was analyzed by Kaplan-Meier analysis.

**Results:**

AC suppressed gastric cancer cell viability and restrained cell migration. AC inhibited the expressions of the cell proliferation marker PCNA and EMT-related marker N-cadherin and increased E-cadherin expression. AC elevated the levels of GRP78 and Chop and suppressed the level of hnRNPA1. In addition, AC restrained gastric cancer proliferation and migration ability and induced endoplasmic reticulum stress by upregulating miR-635 expression. Furthermore, HMGA1 was proven to be a target of miR-635. AC constrained gastric cancer cell proliferation and migration and promoted endoplasmic reticulum stress by regulating HMGA1. Moreover, AC suppressed *in vivo* tumor growth and improved the survival time of mice. Additionally, AC elevated the expressions of miR-635, E-cadherin, GRP78, and Chop and inhibited Ki-67, HMGA1, N-cadherin, and hnRNPA1 expressions in tumor tissues of mice.

**Conclusion:**

AC suppressed gastric cancer progression and induced endoplasmic reticulum stress via the miR-635/HMGA1 axis, providing a valuable drug against gastric cancer.

## 1. Introduction

Gastric cancer is a prevalent malignant tumor globally [1]. The incidence of gastric cancer ranks fifth among all malignancies in recent years, and over 1,000,000 cases are diagnosed each year in the world [2, 3]. At present, surgical resection is the common therapeutic strategy for patients at an early stage [1]. Nevertheless, the majority of patients are diagnosed at the advanced stage [4]. Surgical resection combined with system chemotherapy is the conventional strategy for advanced patients [1]. However, this therapeutic strategy is unsatisfactory, and the prognosis remains poor [5]. Hence, it is imperative to search promising therapeutic strategies against gastric cancer.

In recent years, active compounds from herbal plants are extensively used in the treatment of diseases [6]. Asiaticoside (AC) is the critical compound extracted from *Centella asiatica* [7]. Accumulating evidence reveals that AC exerts vital functions in many diseases [8–11]. For example, AC alleviates the RANKL-induced osteoclastogenesis through modulating NFATc1 and NF-*κ*B signaling pathways [8]. AC also exerts anti-inflammation function to attenuate transient cerebral ischemia-reperfusion injury and allergic response [9, 10]. Besides, AC alleviates bleomycin-induced pulmonary fibrosis through activation of the Cyclic Adenosine Monophosphate (cAMP) and Ras-proximate-1 (Rap1) signaling pathway [11]. Recently, the antitumor role of AC in some cancers has emerged [12–15]. Huang et al. proved that AC promoted cancer cell apoptosis and strengthened the anticancer activity of vincristine [12]. Besides, AC suppressed cell proliferation, migration, and invasion in multiple myeloma drug-resistant cancer [13]. Zhou et al. showed that AC inhibited cell proliferation via regulating the NF-*κ*B pathway in colorectal cancer [14]. Ma et al. demonstrated that AC suppressed cell proliferation and chemotherapeutic drug resistance in hepatocellular carcinoma [15]. However, the effect of AC on gastric cancer progression remains elusive.

MicroRNAs (miRNAs) belong to the small noncoding RNAs family, which usually function as regulators in many diseases via restraining the target gene expression [16]. For instance, miR-142-3p suppresses stem cell properties and enhances sensitivity to radiotherapy via regulating *β*-catenin in breast cancer [17]. miR-378 restrains cell aggressive behaviors through inhibiting SDA1 Domain Containing 1 (SDAD1) in colon cancer [18]. Besides, the regulatory roles of miRNAs in gastric cancer have been reported. Lei et al. showed that miR-143 and miR-145 repressed gastric cancer metastasis via inhibiting Myosin VI (MYO6) expression [19]. miR-206 suppressed gastric cancer cell proliferation via inhibiting cyclinD2 expression [20]. As a member of miRNAs, miR-635 is involved in the regulation of cancers. Tian et al. reported that miR-635 suppressed osteosarcoma progression via inducing cell apoptosis [21]. Zhang et al. discovered that miR-635 repressed tumorigenesis in non-small-cell lung cancer [22]. Besides, miR-635 is decreased in gastric cancer and regulates gastric cancer progression [23]. However, it is unclear whether miR-635 mediates the modulation of AC on gastric cancer development.

Hence, the study focused on investigating the action of AC on gastric cancer progression, determining whether miR-635 participated in the regulation process, and studying the precise mechanism.

## 2. Materials and Methods

### 2.1. Cell Culture

Human normal gastric mucosal epithelium cells GES-1, gastric adenocarcinoma cells AGS, and lymphatic metastatic gastric cancer cells HGC27 were acquired from the Cell Bank of Type Culture Collection of the Chinese Academy of Sciences (Shanghai, China). AGS and HGC27 cells were maintained in RPMI-1640 medium plus 10% FBS (Gibco, NY, USA) at 37°C with 5% CO_2_. At indicated experiments, cells were incubated with AC (0, 0.5, 1, 2, 4, or 8 *μ*M) (Sigma, St. Louis, MO, USA) for 24 h.

### 2.2. Cell Counting Kit-8 (CCK-8) Assay

Cells were inoculated into 96-well plates (2 × 10^3^/well) and cultured for 24 h. Subsequently, 10 *μ*L of CCK-8 (Sigma, St. Louis, MO, USA) was mixed to cells for 2 h of incubation. The optical density was tested by a SpectraMax microtiter plate reader (Molecular Devices, Carlsbad, CA, USA) at 450 nm to analyze cell viability.

### 2.3. Transwell Migration Assay

Cells were treated with 5 *μ*g/mL mitomycin C (Sigma, St. Louis, MO, USA) for 2 h to arrest cell proliferation. Cells were then inoculated into the top chamber of Transwell and maintained in the medium without serum. The bottom chamber was supplied with complete medium. Cells were maintained at 37°C with 5% CO_2 f_or 24 h. The migrated cells were immobilized using 4% paraformaldehyde, dyed using 0.1% crystal violet, and measured utilizing the light microscope.

### 2.4. Western Blot

Cell lysates of AGC and HGC27 cells were extracted using RIPA and quantified using the BCA method. Samples (40 *μ*g) were subjected to SDS-PAGE and electrotransferred onto the PVDF membranes. The membranes were prevented from nonspecific binding and probed with anti-proliferating cell nuclear antigen (PCNA) (ab265585, 1 : 1000), E-cadherin (ab231303, 1 : 1000), N-cadherin (ab98952, 1 : 2000), GRP78 (ab21685, 1 : 1000), Chop (ab11419, 1 : 500), Heterogeneous Nuclear Ribonucleoprotein A1 (hnRNPA1) (ab5832, 1 : 1000), High Mobility Group AT-Hook 1 (HMGA1) (ab129153, 1 : 10000), and GAPDH (ab110305, 1 : 5000) antibodies (Abcam, Cambridge, MA, UK) at 4°C overnight. The membranes were labeled with IgG H&L (HRP) (Abcam, Cambridge, MA, UK). The bands were detected using ECL Plus Western Blotting Substrate (Thermo Fisher Scientific, Waltham, MA, USA), and the density was measured by using ImageJ. GAPDH was designated as the control protein.

### 2.5. Quantitative Real-Time Polymerase Chain Reaction (qRT-PCR)

Total RNAs of AGC and HGC27 cells were obtained utilizing TRIzol, which were used to produce cDNA following the manufacturer's instruction of the TaqMan MicroRNA Reverse Transcription Kit (Thermo Fisher Scientific, Waltham, MA, USA). The qPCR assay was conducted using SYBR Green Master Mix (Thermo Fisher Scientific, Waltham, MA, USA). The PCR conditions were as follows: 95°C for 2 min, 40 cycles of 95°C for 15 sec, and 60°C for 1 min. The primer sequences of miR-635 were as follows: F, 5′-TATAGCATATGCAGGGTG-3′, and R, 5′-CGCATTCGGAGTGCGAGTT-3′ [24]. *U6* served as the control gene. The miR-635 expression was determined utilizing the 2^−ΔΔCt^ method.

### 2.6. Cell Transfection

The inhibitor of miR-635 (anti-miR-635), miR-635 mimic, HMGA1 overexpression plasmid, and their negative controls were acquired from GenePharma (Shanghai, China). The anti-miR-635, miR-635 mimic, or HGMA1 overexpression plasmid were transfected into AGC and HGC27 cells using Lipofectamine 3000 (Invitrogen, Waltham, MA, USA) in the manner of the supplier's protocols. The transfected cells were utilized to conduct subsequent experiments 48 h later.

### 2.7. Bioinformatics Analysis

The possible target gene of miR-635 was screened by bioinformatics analysis using the TargetScan database (http://www.targetscan.org).

### 2.8. Luciferase Assay

The wild and mutant 3′-UTR of HGMA1 was constructed to the psiCHECK-2 plasmid (Promega, Madison, WI, USA). The fused vector and miR-635 mimic or miRNA negative control were cotransfected into AGC or HGC27 cells using Lipofectamine 3000. After 48 h, the luciferase activity was tested utilizing the dual-luciferase reporter assay system (Promega, Madison, WI, USA).

### 2.9. Xenograft Tumor Model

Ten BALB/c nude mice aged 4-6 weeks were bought from Beijing Laboratory Animal Research Center (Beijing, China). The mice were allocated into the control and AC groups (*n* = 5). AGS cells (1 × 10^5^ cells) were injected subcutaneously into the left flank of mice. After 1 week of injection, 10 mg/kg AC and each volume saline were administered to mice in the AC group and control group by oral gavage every 2 days, respectively [14]. Tumor diameters were monitored every week until 5 weeks. Mice were euthanized using CO_2_ inhalation (30% flow rate), and the tumor tissues were collected to conduct qRT-PCR and immunohistochemistry. All mouse experiments complied with the national and international regulations and policies. The study was permitted by the Experimental Animal Ethics of the Second Affiliated Hospital of Wenzhou Medical University.

### 2.10. Immunohistochemistry

Tumor tissues of mice were immobilized in 10% formalin, embedded in paraffin, and made into 4 *μ*m sections. After deparaffinization, rehydration, blocking endogenous peroxidase activity, and nonspecific binding, the sections were treated with anti-Ki-67 (ab264429, 1 : 200), E-cadherin (ab231303, 1 : 500), N-cadherin (ab98952, 1 : 500), GRP78 (ab21685, 1 : 200), Chop (ab11419, 1 : 200), hnRNPA1 (ab5832, 1 : 5000), and HMGA1 (ab129153, 1 : 500) antibodies (Abcam, Cambridge, MA, UK) at 4°C overnight. After rinsing using PBS, the slices were treated with IgG H&L (HRP) for 1 h and dyed with the DAB (R&D Systems, Minneapolis, MN, USA). The staining images were examined using a microscope.

### 2.11. Statistical Analysis

All data were displayed as mean ± standard deviation (SD). Data analysis was completed utilizing SPSS Statistics 22.0 (SPSS, Chicago, IL, USA). The differences between two groups were assessed using the independent *t*-test. One-way ANOVA determined the differences among multiple groups. The survival rate was analyzed using Kaplan-Meier analysis. *P* value below 0.05 was identified as statistically significant.

## 3. Results

### 3.1. Asiaticoside Restrains Cell Aggressive Behaviors and Promoted Endoplasmic Reticulum Stress in Gastric Cancer

**Figure 1:**
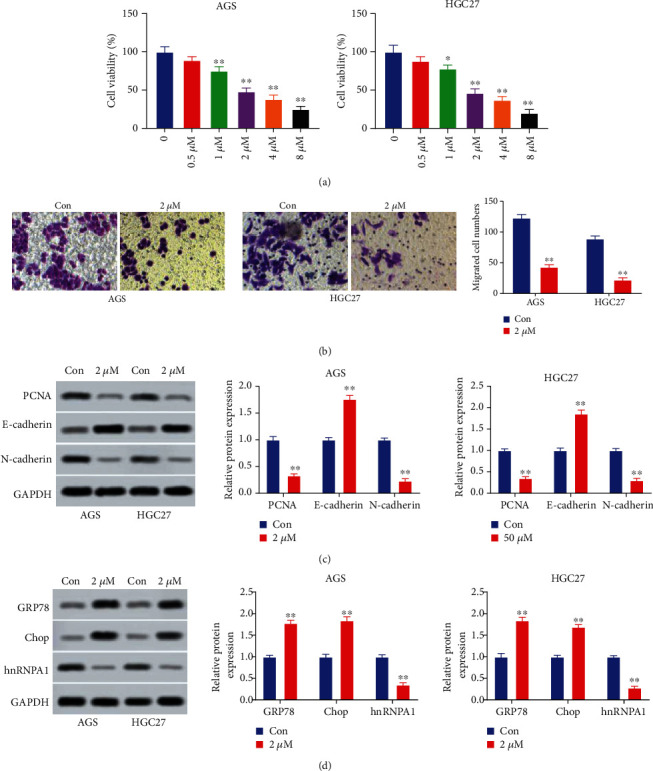
Asiaticoside suppresses aggressive behaviors and induced endoplasmic reticulum stress in gastric cancer. (a) Cell viability of AGS and HGC27 cells after treatment with AC (0, 0.5, 1, 2, 4, or 8) was determined using the CCK-8 assay. (b) Cell migration of cells after treatment with 2 *μ*M AC was measured utilizing the Transwell migration assay. (c) The levels of PCNA, E-cadherin, and N-cadherin in AGS and HGC27 cells after treatment with 2 *μ*M AC were determined utilizing Western blot. (d) The levels of GRP78, Chop, and hnRNPA1 in AGS and HGC27 cells after treatment with 2 *μ*M AC were determined using Western blot. ^∗^*P* < 0.05, ^∗∗^*P* < 0.01.

To investigate the effect of AC on gastric cancer, AGS and HGC27 cells were incubated with AC (0.5 *μ*M, 1 *μ*M, 2 *μ*M, 4 *μ*M, or 8 *μ*M). Cell viability of AGS and HGC27 cells was evaluated after treatment with AC for 24 h. AC decreased cell viability of AGS and HGC27 cells dose-dependently (*P* < 0.05, Figure 1(a)). Besides, the inhibition rate of 2 *μ*M AC on cell viability was 49.03% for AGS cells and 46.82% for HGC27 cells (Figure 1(a)). Therefore, 2 *μ*M AC was used for the subsequent experiments. The Transwell migration assay found that migration ability of AGS and HGC27 cells was suppressed after treatment with 2 *μ*M AC (*P* < 0.01, Figure 1(b)). Additionally, AC inhibited the expressions of PCNA and N-cadherin in both AGS and HGC27 cells (*P* < 0.01, Figure 1(c)). E-cadherin was enhanced after treatment with 2 *μ*M AC (*P* < 0.01, Figure 1(c)). Furthermore, AC remarkably elevated the expressions of GRP78 and Chop and suppressed the level of hnRNPA1 (*P* < 0.01, Figure 1(d)). Therefore, AC suppressed the proliferation and migration ability and induced endoplasmic reticulum stress in gastric cancer.

### 3.2. Asiaticoside Restrains Gastric Cancer Cell Aggressive Behaviors and Induced Endoplasmic Reticulum Stress by Upregulating the miR-635 Expression

**Figure 2:**
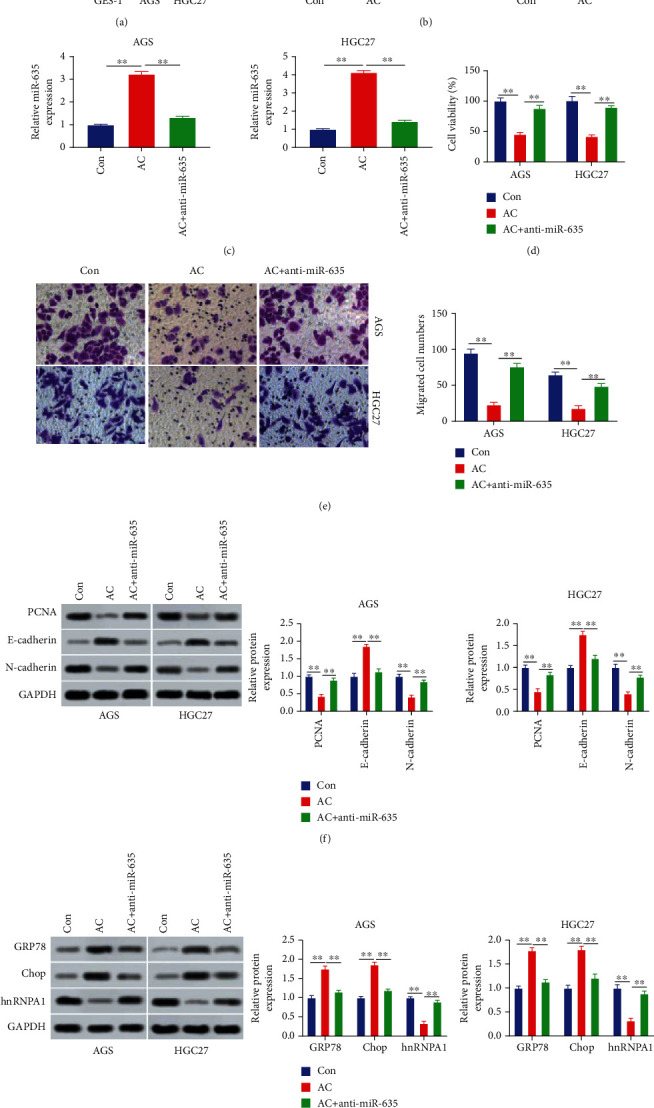
Asiaticoside suppresses gastric cancer aggressive behaviors and induced endoplasmic reticulum stress by upregulating the miR-635 expression. (a) The miR-635 expression in GES-1, AGS, and HGC27 cells was assessed using qRT-PCR. (b) The miR-635 expression in AGS and HGC27 cells after treatment with AC was evaluated. (c) The miR-635 expression was tested utilizing qRT-PCR after cell treatment with AC and transfected with the miR-635 inhibitor. (d) Gastric cancer cell viability was assessed by the CCK-8 assay after AC treatment and miR-635 inhibitor transfection. (e) The migration capacity was evaluated by the Transwell assay after AC treatment and miR-635 inhibitor transfection. (f) The levels of PCNA, E-cadherin, and N-cadherin after AC treatment and miR-635 inhibitor transfection were determined by Western blot. (g) The levels of GRP78, Chop, and hnRNPA1 in AGS and HGC27 cells after treatment with AC and transfection with the miR-635 inhibitor were evaluated using Western blot. ^∗∗^*P* < 0.01.

miR-635 presented lowly expression in gastric cancer and suppressed gastric cancer development [23]. Thus, we explored whether miR-635 mediated the regulation process of AC on gastric cancer aggressive behaviors and endoplasmic reticulum stress. Firstly, miR-635 expression in gastric cancer cells was detected. It was observed that miR-635 was decreased in AGS and HGC27 cells (*P* < 0.01, Figure 2(a)). Then, the influence of AC on miR-635 expression was detected in AGS and HGC27 cells. Results showed that AC considerably elevated the miR-635 expression (*P* < 0.01, Figure 2(b)). After the miR-635 inhibitor was introduced into AGS and HGC27 cells, the promotion effect of AC on miR-635 expression was abolished (*P* < 0.01, Figure 2(c)). Besides, the inhibitory action of AC on cell viability and migration ability was reversed by downregulated miR-635 (*P* < 0.01, Figures 2(d) and 2(e)). Furthermore, the inhibitory effect of AC on the expressions of PCNA and N-cadherin was abrogated upon miR-635 silencing (*P* < 0.01, Figure 2(f)). Instead, the expression of E-cadherin induced by AC was suppressed by downregulated miR-635 (*P* < 0.01, Figure 2(f)). Moreover, the promotion role of AC in GRP78 and Chop expression levels and the inhibitory effect of AC on hnRNPA1 expression were abolished by decreased miR-635 (*P* < 0.01, Figure 2(g)). Thus, AC inhibited gastric cancer cell proliferation and migration and promoted endoplasmic reticulum stress by upregulating miR-635.

### 3.3. HMGA1 Is a Target of miR-635

**Figure 3:**
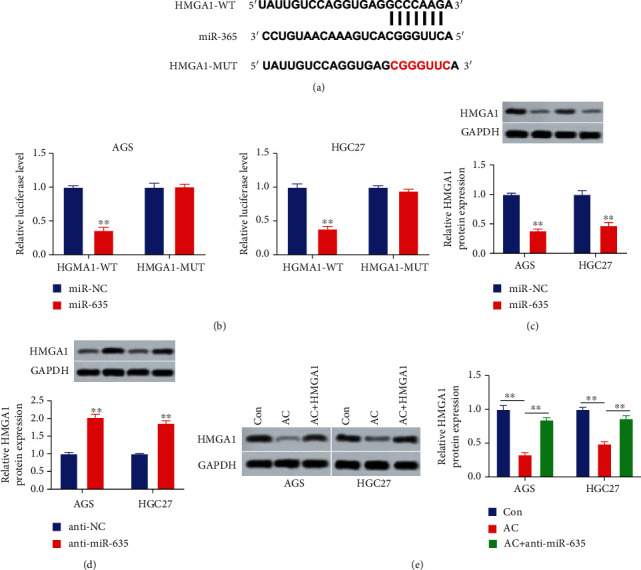
HMGA1 is a target of miR-635. (a) The potential targeting sites of miR-635 and HMGA1 were listed. (b) The target relationship between miR-635 and HMGA1 was testified using the luciferase assay. (c) The HMGA1 level in AGS and HGC27 cells after miR-635 mimic transfection was determined utilizing Western blot. (d) The HMGA1 level in cells with miR-635 inhibitor transfection was assessed using Western blot. (e) The level of HMGA1 in cells after AC treatment and miR-635 inhibitor transfection was evaluated using Western blot. ^∗∗^*P* < 0.01.

Given that miRNAs usually exerted regulation functions through modulating the expressions of target genes, we next screened the target genes of miR-635 in the TargetScan database and found that HMGA1 was targeted by miR-635. The putative targeting sequences between miR-635 and HMGA1 are listed in Figure 3(a). To deeply testify the relationship between miR-635 and HMGA1, the luciferase assay was performed. Overexpressed miR-635 suppressed the luciferase activity of the psiCHECK-2-HMGA1-3′-UTR reporter but had no effect on the psiCHECK-2-MUT-HMGA1-3′-UTR reporter (*P* < 0.05, Figure 3(b)). HMGA1 protein level was suppressed by overexpressed miR-635 and increased by downregulated miR-635 (*P* < 0.01, Figures 3(c) and 3(d)). Furthermore, AC treatment significantly suppressed the HMGA1 protein level which was reversed by downregulated miR-635 (*P* < 0.01, Figure 3(e)). Hence, HMGA1 was a target of miR-635.

### 3.4. Asiaticoside Suppresses Gastric Cancer Cell Aggressive Behaviors and Induced Endoplasmic Reticulum Stress by Regulating HMGA1

**Figure 4:**
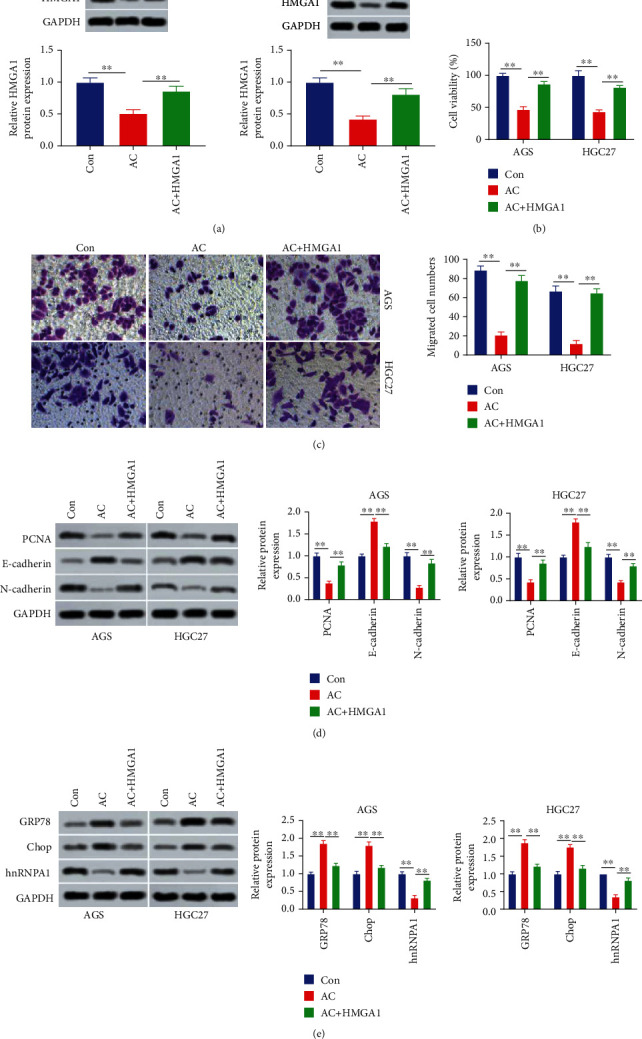
Asiaticoside suppressed gastric cancer cell aggressive behaviors and induced endoplasmic reticulum stress by regulating HMGA1. (a) HMGA1 expression was tested using Western blot in AGS and HGC27 cells after treatment with AC and transfection with the HMGA1 overexpression plasmid. (b) The cell viability after AC treatment and HMGA1 overexpression was assessed by the CCK-8 assay. (c) The cell migration ability of AGS and HGC27 cells after treatment with AC and transfection with the HMGA1 overexpression plasmid was determined using the Transwell assay. (d) The levels of PCNA, E-cadherin, and N-cadherin in cells after AC treatment and HMGA1 overexpression were assessed using Western blot. (e) The levels of GRP78, Chop, and hnRNPA1 in AGS and HGC27 cells after treatment with AC and transfection with the HMGA1 overexpression plasmid were tested using Western blot. ^∗∗^*P* < 0.01.

To determine whether HMGA1 participated in the modulation of AC on gastric cancer cell proliferation, migration, and endoplasmic reticulum stress, the HMGA1 overexpression plasmid was transfected into AC-treated AGS and HGC27 cells. Results showed that AC suppressed the HMGA1 expression, which was abrogated by HMGA1 overexpression plasmid (*P* < 0.01, Figure 4(a)). Besides, AC suppressed cell viability and migration of AGS and HGC27 cells, but they were increased by overexpressed HMGA1 (*P* < 0.01, Figures 4(b) and 4(c)). Furthermore, AC inhibited the levels of PCNA and N-cadherin and increased E-cadherin level, which was reversed by overexpressed HMGA1 (*P* < 0.01, Figure 4(d)). Moreover, AC increased the expressions of GRP78 and Chop and inhibited hnRNPA1 expression, which was abolished by overexpressed HMGA1 (*P* < 0.01, Figure 4(e)). Taken together, AC restrained gastric cancer cell aggressive behaviors and promoted endoplasmic reticulum stress by regulating HMGA1.

### 3.5. Asiaticoside Abates Tumor Growth *In Vivo*

**Figure 5:**
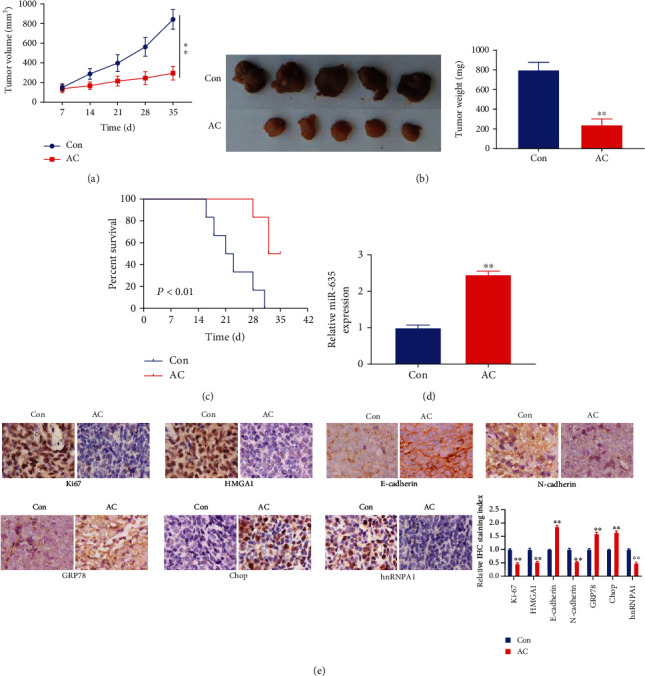
Asiaticoside abated tumor growth *in vivo.* (a) The tumor volumes of mice with or without AC administration were measured every 7 days until 35 days. (b) Images of the xenograft tumors and tumor weights from all mice at the endpoint were presented. (c) The survival rate of mice with or without AC administration was determined using Kaplan-Meier analysis. (d) The miR-635 expression in the tumor of mice with or without AC administration was detected using qRT-PCR. (e) The levels of Ki-67, HMGA1, E-cadherin, N-cadherin, GRP78, Chop, and hnRNPA1 in the tumor of mice with or without AC administration were evaluated by immunohistochemistry. ^∗∗^*P* < 0.01.

To evaluate the antitumor effect of AC on gastric cancer, the xenograft tumor model was established by subcutaneously injecting AGS cells (1 × 10^5^ cells) to nude mice. After 1 week, mice in the AC group and control group orally received 10 mg/kg AC and each volume saline every 2 days, respectively. Results showed that the tumor volume in the AC group was significantly smaller than that in control group from the 14th day (*P* < 0.01, Figure 5(a)). Tumor weight presented obvious suppression after treatment with AC (*P* < 0.01, Figure 5(b)). Kaplan-Meier analysis results indicated that the mice in the AC group exhibited longer survival times than those in the control group (Figure 5(c)). The median survival time in the AC group and control group was 32 and 21 days, respectively (Figure 5(c)). Besides, miR-635 expression was elevated in tumor tissues of mice in the AC group (*P* < 0.01, Figure 5(d)). Furthermore, AC administration suppressed the expressions of Ki-67, HMGA1, N-cadherin, and hnRNPA1 and increased the expressions of E-cadherin, GRP78, and Chop in tumor tissues (*P* < 0.01, Figure 5(e)). Collectively, AC abated tumor growth of gastric cancer.

## 4. Discussion

Gastric cancer is a common malignance accompanied by high morbidity and poor prognosis [1–3, 5, 25]. Therefore, it is pressing to discover curative therapeutic strategies against gastric cancer. AC participates in the regulation of some cancers such as multiple myeloma, colorectal cancer, and colorectal cancer [13–15]. Thus, we conjectured that AC may modulate gastric cancer progression.

To investigate the influence of AC on gastric cancer progression, gastric cancer cells were treated with AC. The gastric cancer cell viability and migration were reduced after treatment with AC. Besides, AC suppressed the expressions of the cell proliferation marker PCNA and EMT-related marker N-cadherin and increased the expression of the EMT-related marker E-cadherin. In other words, AC restrained gastric cancer cell proliferation, migration, and EMT. These findings were in keeping with the reported studies [13–15]. Zhou et al. proved that AC inhibited the cell proliferation ability of colorectal cancer cells [14]. Ma et al. found that AC restrained the proliferation capacity of hepatocellular carcinoma [15]. AC also inhibited multiple myeloma cell aggressive behaviors [13]. Besides, He et al. confirmed that AC repressed the EMT process of pancreatic cancer [26]. Furthermore, many studies demonstrate that endoplasmic reticulum stress exists in gastric cancer, and blocking endoplasmic reticulum stress suppresses gastric cancer cell aggressive behaviors [27–29]. For example, Liu et al. found that YAP accelerated gastric cancer development through regulating endoplasmic reticulum stress [27]. Therefore, we studied the action of AC on the endoplasmic reticulum stress in gastric cancer and proved that AC strengthened the levels of GRP78 and Chop and suppressed the hnRNPA1 level. That is to say, AC induced endoplasmic reticulum stress in gastric cancer. Similarly, Gurfinkel et al. discovered that AC induced endoplasmic reticulum disorder and calcium homeostasis dysregulation during inducing cell apoptosis in prostate cancer cells [30]. Thus, these findings revealed that AC suppressed the cell proliferation and migration ability and EMT and induced endoplasmic reticulum stress *in vitro* in gastric cancer. To better explore the action of AC on gastric cancer progression, we determined the influence of AC on tumor growth *in vivo.* Results indicated that AC treatment suppressed tumor growth and improved mouse survival. AC also suppressed the expression of Ki-67, HMGA1, N-cadherin, and hnRNPA1 and enhanced the expression of E-cadherin, GRP78, and Chop in tumor tissues. In other words, AC inhibited cancer proliferation, migration, and EMT and induced endoplasmic reticulum stress *in vivo*. These results were in harmony with the study performed by Zhou et al. [14]. They found that AC inhibited colorectal tumor growth dose-dependently [14]. Hence, AC suppressed tumor growth of gastric cancer *in vivo*. Together, AC suppresses gastric cancer progression and induces endoplasmic reticulum stress *in vitro* and *in vivo*.

miR-635 is an important cancer regulator in cancers including osteosarcoma, non-small-cell lung cancer, and nasopharyngeal carcinoma [21, 22, 31]. However, there are few articles on the role of miR-635 in gastric cancer, with only Cao et al. finding that miR-635 is decreased in gastric cancer and modulates gastric cancer progression via regulating KIFC1 expression [23]. However, its function on the modulation of AC on gastric cancer development remained elusive. Therefore, we determined whether miR-635 participated in this process. Results indicated that miR-635 was decreased in gastric cancer cells, but AC treatment increased its expression. The inhibitory influences of AC on cell proliferation, migration, and EMT were abrogated by downregulated miR-635. Besides, the promotion effect of AC on endoplasmic reticulum stress was suppressed by downregulated miR-635. The effect of miR-635 on gastric cancer progression was consistent with the previous study [23]. Up to now, this study first reported that AC suppressed gastric cancer cell aggressive behaviors and induced endoplasmic reticulum stress through upregulating miR-635 expression.

miRNAs usually play regulation roles via controlling gene expression [16]. Therefore, the target genes of miR-635 were searched. Among the target genes of miR-635, HMGA1, a small nuclear protein belonging to the HMGA family, is an oncofoetal gene and regulates genesis and development of various neoplasms [32]. Interestingly, HMGA1 displays a critical role in the detection and progression of gastrointestinal tumors [33]. HMGA family members are considered reliable biomarkers able to efficiently track a gastrointestinal tumor in the future [33]. Therefore, HMGA1 was chosen as the target of miR-635 for follow-up study. Results indicated that HMGA1 was targeted by miR-635, and HMGA1 expression was inversely modulated by miR-635. Besides, AC suppressed the HMGA1 protein level, which was reversed by downregulated miR-635. Previous studies showed that HMGA1 functioned as an oncogene and promoted gastric cancer progression [34, 35]. Therefore, we inferred that HMGA1 might exert function in the modulation of AC on gastric cancer progression. To verify the hypothesis, the gastric cancer cells were treated with AC and transfected with the HMGA1 plasmid to change the expression of HMAG1. Afterward, results revealed that the inhibitory influences of AC on cell proliferation, migration, and EMT were reversed by overexpressed HMAG1. Furthermore, the promotion effect of AC on endoplasmic reticulum stress was suppressed by overexpressed HMAG1. The effects of HMAG1 on gastric cancer were in harmony with the published studies. Akaboshi et al. reported that overexpressed HMAG1 maintained the cell proliferation ability of gastric cancer cells [36]. Cao et al. found that overexpressed HMAG1 increased gastric cancer cell proliferation, migration, and invasion ability [34]. Jin et al. revealed that HMGA1 potentiated the aggressive development of gastric cancer via inducing EMT [35]. Therefore, these results indicated that AC suppressed gastric cancer cell aggressive behaviors and induced endoplasmic reticulum stress by regulating HMGA1 expression.

## 5. Conclusion

Asiaticoside suppressed gastric cancer cell aggressive behaviors and stimulated endoplasmic reticulum stress via regulating the expressions of miR-635 and HMGA1. Besides, asiaticoside restrained tumor growth *in vivo*. Therefore, asiaticoside may be a valuable drug against gastric cancer.

## Data Availability

The data used to support the findings of this study are included within the article.
